# Country characteristics and acute diarrhea in children from developing nations: a multilevel study

**DOI:** 10.1186/s12889-015-2120-8

**Published:** 2015-08-21

**Authors:** Ángela María Pinzón-Rondón, Carol Zárate-Ardila, Alfonso Hoyos-Martínez, Ángela María Ruiz-Sternberg, Alberto Vélez-van-Meerbeke

**Affiliations:** Escuela de Medicina y Ciencias de la Salud, Universidad del Rosario, Bogotá, Colombia; Facultad de Medicina, Universidad del Rosario sede Quinta de Mutis, Carrera 24 #63C-69, Bogotá, Colombia

## Abstract

**Background:**

Each year 2.5 billion cases of diarrheal disease are reported in children under five years, and over 1,000 die. Country characteristics could play a role on this situation. We explored associations between country characteristics and diarrheal disease in children under 5 years of age, adjusting by child, mother and household attributes in developing countries.

**Methods:**

This study included 348,706 children from 40 nations. We conducted a multilevel analysis of data from the Demographic and Health Surveys and the World Bank.

**Results:**

The prevalence of acute diarrhea was 14 %. Country inequalities (OR = 1.335; 95 % CI 1.117–1.663) and country’s low income (OR = 1.488; 95 % CI 1.024–2.163) were associated with diarrhea, and these country characteristics changed the associations of well-known determinants of diarrhea. Specifically, living in poor countries strengthens the association of poor household wealth and mother’s lack of education with the disease. Other factors associated with diarrhea were female sex of the child (OR = 0.922; 95 % CI 0.900–0.944), age of the child (OR = 0.978; 95 % CI 0.978–0.979), immunization status (OR = 0.821; 95 % CI 0.799–0.843), normal birthweight (OR = 0.879; 95 % CI 0.834–0.926), maternal age (OR = 0.987; 95 % CI 0.985–0.989), lack of maternal education (OR = 1.416; 95 % CI 1.283–1.564), working status of the mother (OR = 1.136; 95 % CI 1.106–1.167), planned pregnancy (OR = 0.774; 95 % CI 0.753–0.795), a nuclear family structure (OR = 0.949; 95 % CI 0.923–0.975), and household wealth (OR = 0.948; 95 % CI 0.921–0.977).

**Conclusions:**

Inequalities and lack of resources at the country level in developing countries -but not health expenditure- were associated with acute diarrhea, independently of child, family and household features. The broad environment considerably modifies well-known social determinants of acute diarrhea and public health campaigns designed to target diarrhea should consider macro characteristics of the country.

## Background

In spite of global efforts to improve child health, millions of children under the age of five die mostly from preventable causes, including 6.6 million in 2012 [1]. The majority of these deaths occurred in developing countries, predominantly in Asia, Africa and Latin America [2]. Pneumonia is the leading cause of death in this age group, followed by diarrheal disease, which causes 9 % of the fatalities [1]. Each year 2.5 billion cases of diarrheal disease are reported in children under 5 years, and on average every day over 1,400 children die [1, 2]. According to UNICEF and the World Health Organization (WHO), the fight against pneumonia and diarrhea, along with nutritional reinforcement, could save millions of children [3]. In developed nations mortality secondary to diarrhea in this age group is very low and the disease’s great economic cost is the main concern. In contrast, in developing countries, diarrhea’s burden is mainly the loss of human capital due to its high mortality rate [4]. The control of diarrheal disease is imperative in order to decrease mortality in children under 5 years of age and achieve development goals [3]. Information on the disease is needed in order to develop mechanisms to decrease its morbidity and mortality.

A review of factors associated with acute diarrhea was conducted searching in two electronic databases, PubMed and EMBASE. The search methodology is included in Appendix 1. Individual, family and household characteristics have been implicated in the incidence of diarrhea [3, 5, 6]. Most of these associations have been established through studies developed primarily in industrialized nations [5] or limited to specific geographic regions [7–11]. Following Bronfenbrenner’s ecological model, the factors that have been associated with diarrheal disease by individual characteristics and environmental systems are presented below [12]. The child factors that have been associated with diarrhea are young age [11], sex [10], absence of, or short term breastfeeding [6, 9, 11], incomplete immunization schedule [6, 9], moderate to severe undernutrition [6, 9, 11], lack of access to health care [3, 9], and low birthweight [6]. The family and household characteristics that have been related to diarrhea are lack of maternal education [3], maternal employment [3, 9], lack of sanitation [3, 9–11], nontraditional family structures [10], young maternal age [10], poverty [3], residence in rural areas [3], and household overcrowding [3]. Finally, researchers have found heterogeneity across countries in regards to the prevalence of diarrhea, suggesting that the social and economic context at the country level play a role in the incidence of the disease [13].

This paper explores, through multilevel methods, how country characteristics in developing countries from all geographic areas may be fundamental determinants of diarrheal disease, adjusting for known individual, family and household characteristics. It presents the association of country’s wealth (per capita GDP), income inequality (GINI coefficient) and health expenditure, with diarrheal disease in children under 5 years of age from 40 developing countries.

## Methods

### Data sources

We designed a cross-sectional, transnational and multilevel study that used level-1 data (child, mother and household characteristics) from the Demographic and Health Survey (DHS) phase-V [14] and level-2 data (country characteristics) from the World Bank (WB) country data [15].

The DHS phase-V collected data from 41 developing countries from 2004 to 2010. A nationally representative, probabilistic sample including rural and urban areas was collected from each participating country. Respondents were selected through a multistage, stratified sampling procedure of households. Between 5,000 and 30,000 households were surveyed per country. Data was gathered for the following countries: Albania 2008–2009, Azerbaijan 2006, Bangladesh 2007, Benin 2006, Bolivia 2008, Cambodia 2010, Colombia 2010, Congo 2005, Egypt 2005–2006, Philippines 2008, Ghana 2008, Guyana 2009, Haiti 2005–2006, Honduras 2005–2006, India 2005–2006, Indonesia 2007, Jordan 2007, Kenya 2008–2009, Lesotho 2009, Liberia 2007, Madagascar 2008–2009, Malawi 2010, Maldives 2009, Mali 2006, Namibia 2006–2007, Nepal 2006, Niger 2006, Nigeria 2008, Pakistan 2006–2007, Peru 2004–2008, Democratic Republic of Congo 2007, Dominican Republic 2007, São Tomé é Príncipe 2008–2009, Sierra Leone 2008, Swaziland 2006–2007, Tanzania 2010, East Timor 2009–2010, Ukraine 2007, Uganda 2006, Zambia 2007 and Zimbabwe 2005–2006. We excluded Ukraine from the analysis because the country did not apply the child health module of the survey. Information from the remaining 40 countries was merged to create a single dataset, which included 395,485 households with children. The dataset was further limited to biological mothers answering the survey to assure comparability (384,662), living children (359,527), permanent household residents (349,849) and cases with complete information (348,706).

After careful analysis, we concluded that the WB country data was the best source of level-2 data in this study because of its country comparability and robustness when compared to data from other sources. These data included the 2010 indicators: per capita gross domestic product (GDP), Gini-coefficient, and health expenditure as a percentage of GDP.

### Outcome measures

**Diarrheal disease**: presence of diarrhea (as defined by the respondent, the child’s mother) at any time during the 2 weeks preceding the interview (0 = no; 1 = yes). Diarrhea was defined by DHS to the mothers as increased frequency of depositions and/or low consistency of feces. DHS does not distinguish by severity or number of episodes.

### Variables

We divided the variables according to the data source: level-1 variables included the child, mother and household characteristics, and level-2 variables included country data. Initially we considered three levels of analysis –child, household and country- but most households had only one child under the age of five, so it was decided to include only one child per household, the youngest, and conduct a two-level analysis.

**Level-1 data, children: sex** coded as 0 = male and 1 = female, **age** of child in months, **immunization** defined as completeness of WHO schedule, coded as 0 = incomplete and 1 = complete, **duration of breast feeding** in months, **possession of health card** coded as 0 = no and 1 = yes, **undernutrition** defined as a BMI (body mass index) under the 5th percentile, and **birthweight** codified in dummy variables as follows: normal -above 2,500 g- 0 = no, 1 = yes; low -below 2500 g- 0 = no, 1 = yes; and no weighted, 0 = no, 1 = yes. Although it is not ideal to include a birthweight missing indicator, taking into consideration that 46 % of the children did not have this information, imputation was ruled out.

**Level −1 data, mother: age** in years, **education**: no education coded as 0 = no, 1 = yes; elementary education coded as 0 = no, 1 = yes; high school education coded as 0 = no, 1 = yes; superior [technical or professional] education coded as 0 = no, 1 = yes), **current employment** coded as 0 = no and 1 = yes and **planned pregnancy** referring to the pregnancy in which the index children was the outcome and coded as 0 = no; 1 = yes.

**Level −1 data, household: number of household members** defined as number of people living in the same home, **place of residence** coded as 0 = rural and 1 = urban, **nuclear family** defined as a social unit conformed exclusively by two parents and one or more children: 0 = non-nuclear family, 1 = nuclear family, **sanitation** score based upon water source and waste disposal, both classified as improved or unimproved, from zero to two, with higher grades indicating better sanitation [16], and **wealth index** calculated by the DHS considering income, possessions and quality of life, with higher grades indicating greater wealth. Wealth index ranges from 1 to 5.

**Level-2 data, country: Country wealth** coded as a set of dummy variables as follows: Low income (1 = gross domestic product per capita (GDPpc) of US$1,025 or less), Lower middle income (1 = GDPpc between US$1,026 and US$4,035), Upper middle income (1 = GDPpc between $4,036 and $12,475), and High income (1 = GDPpc of $12,476 or more) [15], **Inequality** based on the Gini coefficient (1 = top 25 % unequal countries and 0 = more equal countries) and **health expenditure** coded as a set of dummy variables based on the percentage of GDP expended on health. Low health expenditure (1 = 5 % or less), Middle health expenditure (1 = between 5.1 and 10 %), High health expenditure (1 = more than 10 %). We considered in the initial models country homicide rates and total country population, but these variables were omitted in the final models because of their lack of association with diarrheal disease and their negative effects on the model’s validity, measured using residual files and reliability estimates.

### Statistical analysis

The analysis was conducted considering known factors associated with diarrhea and the country characteristics to study. Multilevel analyses were preferred because the hierarchical nature of the data violated the principles of independence and homogeneity required for a single-level analysis [17]. Country characteristics were shared by many children.

All the variables were used in the initial analysis. Afterwards, sanitation and number of members in the household were excluded due to co-linearity. The wealth index was built considering both variables. Possession of a health card and undernutrition were excluded for their co-linearity with immunization and wealth index, respectively. Although low BMI has been highly associated with diarrhea, wealth index was used over undernutrition in the models because the former had 35 % of its data missing.

The statistical analysis was performed using SPSS 20.0 (IBM) and HLM 7 (Scientific Software International, Inc.), as follows: 1) we merged the individual datasets of the 40 countries, 2) we calculated descriptive statistics for categorical (proportions) and numerical variables (mean, standard deviation, minimum and maximum values), 3) we obtained bivariate odd ratios using hierarchical linear modeling logistic regressions of diarrheal disease in all of the studied variables, and 4) we generated multivariable models for diarrhea using hierarchical linear modeling. Stepwise multilevel logistic regression equations were estimated. Individual, family and household factors were included as possible predictors of diarrhea, and differences were deemed to be significant with *P*-value less than 0.05. The large sample size allowed us to find small differences with narrow 95 % confidence intervals. Finally, multilevel modeling was used to explore the association of country characteristics with diarrheal disease (between countries associations) adjusting for individual, family and household predictors of the condition (within countries associations) [18, 19]. Full maximum likelihood was used to fit the models. Random effects were estimated only for indicators with variations between groups that could be explained by the studied variables, allowing the coefficients to vary across groups. Those level-1 indicators were centered on the country mean to avoid the problem of co-linearity. All other variables, as well as the neighborhood variables, were centered on the grand mean and we constrained their variance. The final model can be seen in Appendix 2. We have calculated median odd ratios (MORs) and intra-class correlations (ICC) for the models, as well as 80 % interval odd ratios (IORs) for the country level variables [20, 21].

We tested bivariate interactions by multiplying duration of breast feeding and maternal education, duration of breast feeding and maternal employment, immunization and maternal education, and wealth index and immunization to determine if an interaction was present.

Within countries, weights provided by the DHS for children under 5 years of age were utilized in the analysis for the level 1 data. The weights were adjusted to the survey design. Post-stratification was incorporated as a weight adjustment. The adjusted weights were used in all of the analyses. For level 2 data, between countries, weights were created and used in the analysis for each country accounting for the country’s population.

Regression analyses considering the DHS year of survey were performed to assure that the results were not biased by the different time lapses the surveys took place at each country.

Macro International provided the datasets from the 41 countries included in this study. The study was based on secondary sources without identifying information about individual participants. It was given approval by the institutional review board, Comité de Ética en Investigación, Universidad del Rosario.

## Results

### Descriptive statistics

**Fig. 1:**
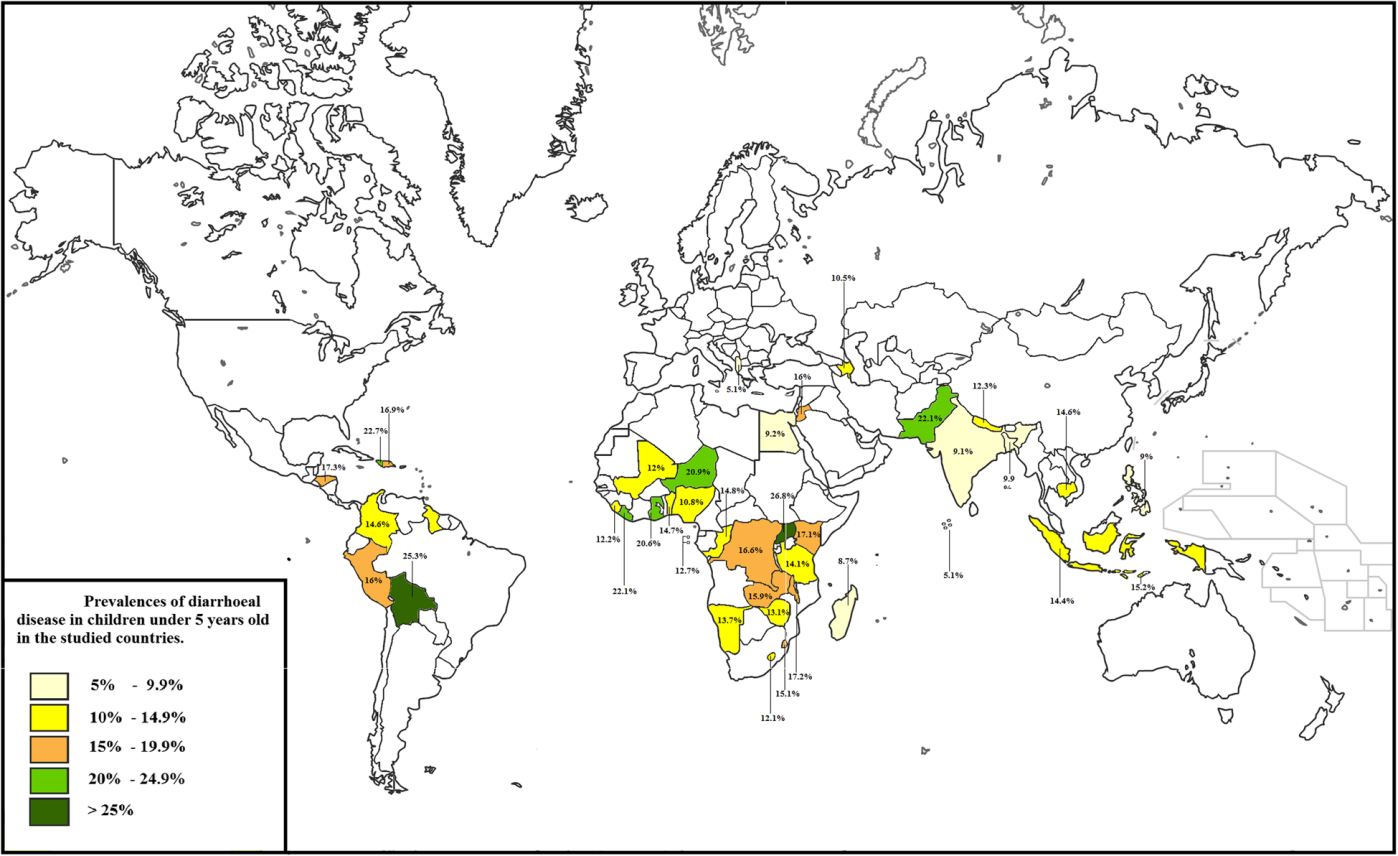
Prevalences of diarrhocal disease in children under 5 years old in the studied countries

The final sample included 348,706 children under 5 years of age from 40 developing nations. Figure 1 presents the prevalence of diarrheal disease in each of the studied countries. The descriptive characteristics of the study population are shown in Table 1. The complete dataset had an even child gender distribution, and the average age of the children was 29 months. The overall prevalence of diarrhea in the 2 weeks preceding the survey was 14 %.

**Table 1:** Descriptive statistics. Proportions of categorical variables and mean/standard deviation of numerical variables - children from 40 countries, 2004–2010

Variable	Proportion	Mean	SD
Children			
Diarrheal disease	0.14		
Female sex	0.49		
Age of child (months)		28.76	17.25
Immunization	0.58		
Possession of health card	0.84		
Undernutrition	0.06		
Normal birth weight	0.48		
Low birth weight	0.06		
No weighed at birth	0.46		
Duration of breastfeeding (months)		13.73	9.86
Mother			
Age of mother (years)		28.66	6.72
No education	0.30		
Elementary education	0.33		
High school education	0.30		
Superior education	0.07		
Employment	0.50		
Planned pregnancy	0.70		
Household			
Number of household members		6.59	3.2
Sanitation		1.24	0.74
Urban residence	0.35		
Nuclear family	0.69		
Wealth index		3.00	1.41
Country data			
Low income	0.48		
Lower middle income	0.35		
Upper middle income	0.18		
Inequalities	0.25		
Low health expenditure	0.33		
Middle health expenditure	0.63		
High health expenditure	0.08		

Only 58 % of the children were up-to-date on their immunization schedule, and 84 % had a health card. Approximately half (48 %) of the children had normal birthweight, and 6 % were undernourished. The children were, on average, breastfed for 14 months.

Almost a third of the mothers did not have education (30 %), half of them were employed, and 70 % reported a planned pregnancy. Most households (65 %) were located in rural areas, with an average number of seven inhabitants, and most of them (69 %) had a nuclear structure.

### Bivariate logistic regressions

Results of the bivariate regression analysis are shown in Table 2.

**Table 2:** Bivariate regressions of acute diarrheal disease on independent variables

Variable	OR	CI	*P*-value
Children			
Female sex	0.923	0.901, 0.946	<0.001
Age (months)	0.978	0.976, 0.981	<0.001
Immunizations	0.824	0.801, 0.838	<0.001
Duration of breastfeeding (months)	0.989	0.985, 0.992	0.026
Possession of health card	0.723	0.604, 0.842	<0.001
Underweight	1.091	1.069, 1.109	<0.001
Infant birth weight			
Normal birth weight	0.904	0.868, 0.923	<0.001
Low birth weight	1.022	1.080, 1.199	0.003
No weighed at birth	1.064	1.046, 1.109	<0.001
Mother			
Age of mother (years)	0.978	0.972, 0.984	<0.001
Maternal education			
No education	1.119	1.086, 1.151	<0.001
Elementary education	1.087	1.056, 1.118	<0.001
High school	0.931	0.901, 0.963	<0.001
Superior education	0.649	0.609, 0.701	<0.001
Employment	1.011	0.984, 1.033	0.632
Planned pregnancy	0.727	0.709, 0.752	<0.001
Household			
Number of household members	1.011	1.007, 1.014	0.002
Urban residence	0.661	0.851, 0.899	<0.001
Nuclear family	0.880	0.861, 0.906	<0.001
Sanitation	0.901	0.887, 0.921	<0.001
Wealth index	0.918	0.911, 0.928	<0.001
Country			
Country level income			
Low income	1.257	1.041, 1.676	<0.001
Lower middle income	0.881	0.653, 1.172	0.400
Upper middle income	0.803	0.562, 1.149	0.096
Inequality	1.232	1.012, 1.609	0.043
Country level health expenditure			
Low health expenditure	0.858	0.641, 1.137	0.295
Middle health expenditure	1.118	0.843, 1.498	0.442
High health expenditure	1.089	0.730, 1.631	0.745

*P* < 0.05, *P* < 0.01, *P* < 0.001, *P* < 0.0001

### Multivariable logistic regressions

The results of logistic regression for diarrheal disease are presented in Table 3. After controlling for all of the study variables we found that both country-level low income and country-level inequality were positively associated with diarrhea. Health expenditure did not have a significant association with diarrhea.

**Table 3:** Adjusted odds ratio (OR) for diarrheal disease associated with individual and country level characteristics among children under 5 years from developing countries calculated using an HLM multivariate multilevel logistic regressions population average fixed effects

	Model 1. Adjusted for children, mother and household characteristics		Model 2. Adjusted for model 1 and for country characteristics		80 % IOR
Reliability	99 %		99 %		
Variables	OR	CI	OR	CI	
Level-1					
Children					
Female sex	**0.923**	[0.902, 0.944]	**0.922**	[0.900, 0.944]	
Age of the child (months)	**0.979**	[0.978, 0.979]	**0.978**	[0.978, 0.979]	
Immunizations	**0.820**	[0.773, 0.870]	**0.821**	[0.799, 0.843]	
Infant birth weight					
Low birth weight	Reference category		Reference category		
Normal weight at birth	**0.881**	[0.838, 0.926]	**0.879**	[0.834, 0.926]	
No weighed at birth	**0.879**	[0.835, 0.926]	**0.879**	[0.834, 0.926]	
Duration of breastfeeding (months)	0.984	[0.982, 1.098]	0.984	[0.966, 1.098]	
Mother					
Age of the mother (years)	**0.987**	[0.986, 0.989]	**0.987**	[0.985, 0.989]	
Maternal Education					
No education of the mother^a^	**1.422**	[1.299, 1.557]	**1.416**	[1.283, 1.564]	
Elementary education	**1.430**	[1.341, 1.524]	**1.453**	[1.357, 1.556]	
High school education	**1.274**	[1.200, 1.353]	**1.286**	[1.205, 1.373]	
Superior education	Reference category		Reference category		
Employment	**1.135**	[1.106, 1.165]	**1.136**	[1.106, 1.167]	
Planned pregnancy	**0.776**	[0.756, 0.797]	**0.774**	[0.753, 0.795]	
Household					
Nuclear family	**0.943**	[0.925, 0.974]	**0.949**	[0.923, 0.975]	
Urban residence	**1.047**	[1.014, 1.081]	1.044	[0.969, 1.079]	
Wealth Index	**0.950**	[0.922, 0.972]	**0.948**	[0.921, 0.977]	
Level – 2					
Country					IOR
Country-level Income					
Low income			**1.488**	[1.024, 2.163]	[1.094, 2.131]
Lower middle income			1.157	[0.786, 1.704]	[0.817,1.629]
Upper middle income			Reference category		
Inequality			**1.335**	[1.117, 1.663]	[0.954, 1.470]
Country-level Health expenditure					
Low health expenditure			Reference category		
Middle health expenditure			1.192	[0.888, 1.601]	[0.916, 1.548]
High health expenditure			1.006	[0.590, 1.715]	[0.989, 0.994]

^a^Since “No maternal education” is involved in an interaction, it is important to clarify that the OR presented in the table is the effect of “No maternal education” in countries with high GDP per cápita, no inequality and low health expenditure

Statistically significant ORs are reported in bold (P values ≤ 0.05)

After data analysis the following child factors were associated with the onset of diarrheal disease: female gender, age, immunization and normal birthweight. The maternal factors negatively associated with diarrhea were age and planned pregnancy. The maternal factors positively associated with diarrhea were lack of education, elementary education and high school education (as compared with superior education) and maternal employment. Household factors negatively associated with the disease were a nuclear family structure and wealth. The between countries variations were evaluated using the variances. Variables that presented statistically significant between-countries variances were immunization status, maternal education, and household wealth. Only the country variances of lack of maternal education on diarrhea (*p*-value <0.001) and household wealth on diarrhea (*p*-value <0.001) were in part explained by the country variables (low income country). All other interactions were non-significant (see Table 4). The Median Odds Ratios (MORs) were 1.51 for model 1 and 1.43 for model 2.

**Table 4:** Interactions between individual and country level variables from multilevel regressions of child, mother, household and country characteristics on diarrheal disease of children under 5 years

	OR	*P* value
“No education of mother” and “Country GDP per capita”		
Low income countries with low health expenditure and high inequalities	1.821	0.025
Middle income countries with low health expenditure and high inequalities	1.368	0.075
Upper middle income countries with low health expenditure and high inequalities	Reference category	
Low income countries with middle health expenditure and high inequalities	1.872	0.026
Middle income countries with middle health expenditure and high inequalities	1.382	0.087
Upper middle income countries with middle health expenditure and high inequalities	Reference category	
Low income countries with upper health expenditure and high inequalities	1.683	0.040
Middle income countries with upper health expenditure and high inequalities	1.224	0.141
Upper middle income countries with upper health expenditure and high inequalities	Reference category	
Low income countries with low health expenditure and low inequalities	1.488	0.032
Middle income countries with low health expenditure and low inequalities	1.143	0.198
Upper middle income countries with low health expenditure and low inequalities	Reference category	
Low income countries with middle health expenditure and low inequalities	1.533	0.031
Middle income countries with middle health expenditure and low inequalities	1.186	0.287
Upper middle income countries with middle health expenditure and low inequalities	Reference category	
Low income countries with upper health expenditure and low inequalities	1.374	0.046
Middle income countries with upper health expenditure and low inequalities	1.107	0.423
Upper middle income countries with upper health expenditure and low inequalities	Reference category	
“Household wealth” and “Country GDP per capita”		
Low income countries with low health expenditure and high inequalities	0.994	0.046
Middle income countries with low health expenditure and high inequalities	0.996	0.235
Upper middle income countries with low health expenditure and high inequalities	Reference category	
Low income countries with middle health expenditure and high inequalities	0.969	0.048
Middle income countries with middle health expenditure and high inequalities	0.981	0.125
Upper middle income countries with middle health expenditure and high inequalities	Reference category	
Low income countries with upper health expenditure and high inequalities	0.980	0.050
Middle income countries with upper health expenditure and high inequalities	0.987	0.257
Upper middle income countries with upper health expenditure and high inequalities	Reference category	
Low income countries with low health expenditure and low inequalities	0.979	0.049
Middle income countries with low health expenditure and low inequalities	0.982	0.279
Upper middle income countries with low health expenditure and low inequalities	Reference category	
Low income countries with middle health expenditure and low inequalities	0.956	0.042
Middle income countries with middle health expenditure and low inequalities	0.972	0.064
Upper middle income countries with middle health expenditure and low inequalities	Reference category	
Low income countries with upper health expenditure and low inequalities	0.966	0.045
Middle income countries with upper health expenditure and low inequalities	0.975	0.128
Upper middle income countries with upper health expenditure and low inequalities	Reference category	

Living in poor and unequal countries increases the magnitude of the association of lack of maternal education with diarrhea. Living in poor countries increases the association between wealth and diarrhea.

When adding the interaction terms at the level 1 to the models, none of them - duration of breast-feeding and maternal education, duration of breast-feeding and maternal employment, immunization and maternal education, and wealth index and immunization- were statistically significant.

## Discussion

This paper contributes to the study of the associations between country characteristics and child health. We found that low income and inequality at the country level, but not health expenditure, were associated with diarrheal disease. These findings suggest that poverty and inequality are associated with child health. Children who live in developing countries with greater per capita GDP and lower inequality were less likely to develop diarrhea. These findings support the importance of fundamental determinants of health, such as per capita GDP and income inequalities, promulgated by social epidemiology [22]. The results could be explained by Bronfenbrenner’s ecological model, which considers the importance of macro level conditions on individual wellbeing [12].

On the contrary, diarrheal disease was not associated with a country’s health expenditure. It is important to interpret this finding considering that child health in general is not improved exclusively by the amount of money a country invests in health. Establishing an association between health expenditure and health is not easy, since it depends on the characteristics of the expenditure as well as on many factors associated with the health outcomes, and it was not possible to consider all of these in this paper [23].

The associations of some of the well-known determinants of health, such as immunization status, maternal education, and household wealth with diarrhea, were considerably affected by country characteristics. The country variables included in the analysis could only partially explain the effect the country level exerts over the relationship of diarrhea with lack of maternal education and household wealth. In richer countries, lack of maternal education and household poverty were not as important as they were in poorer countries. In the same direction, the lack of maternal education had a greater association with diarrheal disease in countries with larger inequality.

These findings support the importance of healthy environments on child health, even if these environments seem distant to the child [24]. The characteristics of the country in which an individual develops, molds him in a several different ways from culture and identity to constraints and possibilities. Poor countries do not have the resources and infrastructure to adequately protect their nationals. Similarly, unequal countries pose a burden on their citizens who perceive the income distribution as unfair. Health interventions often focus on individual-level as a unit of analysis and treat all countries as equal, while the interventions should be tailored to the country’s characteristics. According to our results, inequalities and poverty at the country level should be fought in order to improve child health. Particular consideration should be given to poor and unequal developing countries where poor and uneducated women should receive special programs to prevent child disease.

In this study, child-related characteristics associated with diarrhea were as follows. (1) Gender: Compared to girls, boys had 9 % higher odds of developing diarrhea. This is consistent with the results of another study on diarrhea epidemiology [24]. (2) Age: Each additional month of age decreased on average by 2 % the odds of having diarrhea, and this finding supports what has been reported in the literature [24]. (3) Immunization: Compared with children with complete immunization, those with an incomplete immunization status had 22 % higher odds of developing diarrhea. However, this DHS did not assess vaccination against rotavirus, which could reduce even more the burden of the disease in young children [25, 26]. (4) Birthweight: children born ≥ 2,500 g had 14 % less probability of having diarrhea. A low birthweight could imply maternal nutritional deficiency that has been associated with diarrhea and child mortality [27, 28]. In a 2005–2007 study on diarrhea-related child mortality in the USA, Mehal *et al.* concluded that 86 % of the children who died had a low birthweight [5]. (5) Breast feeding: Bivariate correlation analysis showed a negative association between diarrhea and breast feeding, as has been established in the literature [29]. However, this association was not seen on multivariable models, probably because the mothers had reported the duration of breast-feeding, but not information on simultaneous complementary feeding, and because other variables in the model may have interfered with this association.

The maternal characteristics associated with diarrhea in this study were as follows. (1) Age: Younger mothers reported diarrhea more frequently than older ones, which could be explained in part by the younger mothers’ lack of experience with child care [30]. (2) Maternal education: Compared with children of highly educated mothers, the children of mothers without formal education, with elementary education and with high school education had 42, 45 and 29 % higher odds of having diarrhea, respectively. These findings confirm the results reported in medical literature [31]. (3) Occupation: Children of working mothers had 14 % higher odds to have diarrhea than those of mothers who did not work. This finding supports those who affirm that a mother’s work is detrimental to her child’s well-being. It is probable that the mothers’ absence from the household, along with poor social support had negative effects on child health [32, 33]. (4) Planned pregnancy: We found that the children who were a result of a planned pregnancy had 29 % smaller odds of developing diarrhea than did children who were the result of an unplanned pregnancy. Children from unplanned pregnancies are less likely to receive adequate care [34]. Additionally, family planning decreases the risk of low birth weight [35], which is a known risk factor for diarrhea [5].

The household characteristics associated with diarrhea in this study were as follows. (1) Number of household members: This was associated with diarrhea on bivariate correlation but not on the multivariable model, probably due to other household conditions included in the present study that may be associated with overcrowding. (2) Type of residence: Bivariate correlation showed that children from rural areas were more likely to present with diarrhea, as described previously in the literature [36]. This association disappeared on multivariable models, probably due to the introduction of household wealth in the multivariable models. (3) Nuclear families: Children from nuclear families had 5 % smaller odds of developing diarrhea than did children from non-nuclear families. This outcome is probably secondary to the described effect of social stability that a nuclear family structure has on child health [37]. (4) Sanitation: Adequate sanitation conditions helped prevent diarrhea. It has been estimated that approximately 88 % of diarrhea-induced deaths in the world are attributable to inadequate water supplies, poor hygiene and unsanitary conditions [38]. Moreover, most of these deaths (84 %) occur in children and in developing countries [39]. (5) Wealth index: As expected, children from wealthier families had a lower probability of developing diarrhea. This result is consistent with what has been reported in the literature [22, 40].

### Limitations

DHS have great advantages such as the quality, comparability and representativeness of the information. Nevertheless, the study has some important limitations. First, its cross-sectional nature does not allow establishment of causality. Second, the DHS questionnaires were not performed simultaneously in every country. Although our analysis did not change after controlling for year of survey, social conditions are dynamic and tend to change over time, therefore some differences could be expected. Third, data was collected exclusively from the mother, and while they tend to be the best historian concerning their child’s history, some recall bias is likely. Fourth, the variable definitions are limited by the information available in the DHS. Finally, the large sample size contributes to an over-power analysis that could detect minimal effect sizes, and these effect sizes could be the result of slight biases in the sampling process.

## Conclusions

Diarrhea is a major health problem worldwide, particularly in children living in developing countries. The study of fundamental determinants of health, “determinants of determinants”, could be the foundation upon which we formulate promising solutions that are tailored to the reality of populations and the environment they are immersed. This study explored the association of per capita GDP, income inequalities (Gini coefficient) and health expenditure with diarrheal disease in a large, multinational population adjusting by individual, family and household characteristics, using a multilevel design.

We found a clear association between socio-economic factors and the onset of diarrheal disease in children under 5 years old from developing countries. The factors associated with a greater risk of developing diarrhea are low maternal education, mother employment, poverty and inequality. The factors associated with a lower risk of diarrheal disease are female gender, older children, complete immunization status and normal birthweight. The maternal protective factors were older age and planned pregnancy. The household and country protective factors were a nuclear family structure, appropriate sanitation and greater wealth of the household.

Economic development and inequalities at the country level are contributing to the onset of diarrhea, regardless of children, maternal or household characteristics. The individual features associated with the onset of diarrhea are directly affected by the country’s inequality and poverty. Additionally, health expenditure does not seem to play a key role in the onset of the disease. Hence, in order to effectively address diarrheal disease and child wellbeing in general, these country features (per capita GDP and inequalities) should be taken into consideration.

These results are helpful for initiatives such as the WHO’s Child Health Epidemiology Reference Group in determining factors associated with child morbidity and possible interventions [41].

## Appendix 1. Literature search strategy

The review was conducted searching in two electronic databases (PubMed and EMBASE). 788 studies, written in English and Spanish, were identified from January 1st 2003 until December 31 2014. We used the following MeSH words in PubMed.

#1. MeSH Headings: “Infant”[Mesh] OR “Infant, Newborn”[Mesh] OR “Child, Preschool”[Mesh] OR “Child”[Mesh]

#2. MeSH Subheadings: “Diarrhea/epidemiology”[Mesh] OR “Diarrhea/prevention and control”[Mesh] OR “Diarrhea/complications”[Mesh] “Diarrhea, Infantile/epidemiology”[Mesh] OR “Diarrhea, Infantile/mortality”[Mesh] OR “Diarrhea, Infantile/prevention and control”[Mesh] OR “Diarrhea, Infantile/complications”[Mesh] OR “Gastroenteritis/prevention and control”[Mesh]

#3. MeSH Headings and Subheadings: “Breast Feeding”[Mesh] OR “Risk Factors”[Mesh] OR “Poverty”[Mesh] OR “Developed Countries”[Mesh] OR “Developing Countries”[Mesh] OR “Malnutrition/complications”[Mesh] OR “Malnutrition/prevention and control”[Mesh] OR “Parents/education”[Mesh] OR “Delivery of Health Care/utilization”[Mesh] OR “Health Status Disparities”[Mesh]

#4. #1 AND #2 AND #3

The key words were slightly adapted for EMBASE.

Duplicate records were removed, leaving 485 papers. Subsequently, we screened and evaluated the articles reading their titles and abstracts. After this process we had 94 potentially useful studies based on their assessment of determinants of diarrhea. We read all these articles and picked the best ones to reference in our paper.

After cleaning the data, an additional literature review was done in order to explore the association of each of the available variables with diarrheal disease.

## Appendix 2. Specifications for this Bernoulli HLM2 run

The maximum number of level-1 units = 348,706

The maximum number of level-2 units = 40

The maximum number of micro iterations = 14

Method of estimation: full PQL

Maximum number of macro iterations = 100

Distribution at Level-1: Bernoulli

Weighting Specification

**Table:**

	Weighting?	Weight Variable	Normalized?
Level 1	Yes	SWEIGHT	yes
Level 2	Yes	WEIGHT2	yes
Precision	No		

The outcome variable is Diarrhea

Summary of the model specified

### Level-1 model

Prob(DIARRHEAij = 1|βj) = ϕij

log[ϕij/(1 - ϕij)] = ηij

ηij = β0j + β1j*(Female sex ij) + β2j*(Age of the child ij) + β3j*(Immunizations ij) + β4j*(Normal weight at birth ij) + β5j*(No weighed at birth ij) + β6j*(Duration of breastfeedingij) + β7j*(Age of the mother ij) + β8j*(No education of the mother j) + β9j*(Elementary education ij) + β10j*(High school education ij) + β11j*(Employment ij) + β12j*(Planned pregnancy ij) + β13j*(Nuclear family jj) + β14j*(Urban residence ij) + β15j*(Wealth Index ij) + β16j*(Survey year ij)

### Level-2 model

β0j = γ00 + γ01*(Low income j) + γ02*(Middle income j) + γ03*(Inequality j) + γ04*(Middle health expenditure j) + γ05*(High health expenditure j) + u0j

β1j = γ10

β2j = γ20

β3j = γ30

β4j = γ40

β5j = γ50

β6j = γ60

β7j = γ70

β8j = γ80 + γ81*(Low income j) + γ82*(Middle income j) + γ83*(Inequality j) + γ84*(Middle health expenditure j) + γ85*(High health expenditure j) + u8j

β9j = γ90

β10j = γ100

β11j = γ110

β12j = γ120

β13j = γ130

β14j = γ140

β15j = γ150 γ151*(Low income j) + γ152*(Middle income j) + γ153*(Inequality j) + γ154*(Middle health expenditure j) + γ155*(High health expenditure j) + u15j

β16j = γ160

Immunizations, No maternal education and Wealth index have been centered around the group mean.

All other variables have been centered around the grand mean.

\Level 2 variables have been centered around the grand mean.

## Abbreviations

BMI: Body mass index
CI: Confidence interval
DHS: Demographic and health survey
GDP: Gross domestic product
ICC: Intra-class correlations
IOR: Interval odd ratios
MOR: Median odd ratios
OR: Odds ratio
WB: World Bank
WHO: World Health Organization
